# Supplementary material for: Redox Mechanism of Azathioprine and Its Interaction with DNA
Source: Int J Mol Sci. 2021 Jun 24;22(13):6805. doi: 10.3390/ijms22136805 (PMC8268956; doi:10.3390/ijms22136805)
Supplement: Supplementary file 1 [file ijms-22-06805-s001.zip › ijms-1255133-supplementary.pdf]

Supplementary information

Redox mechanism of azathioprine and its interaction with DNA

Mihaela-Cristina Bunea[1,2], Victor-Constantin Diculescu[1], Monica Enculescu[1], Horia Iovu[2] and Teodor Adrian Enache[1]*

[1]National Institute of Materials Physics, Atomistilor 405A, 077125, Magurele Romania

[2]University Politehnica of Bucharest, Advanced Polymer Materials Group, 1-7 Gh. Polizu Str. Bucharest 011061, Romania

*Corresponding authors: adrian.enache@infim.ro

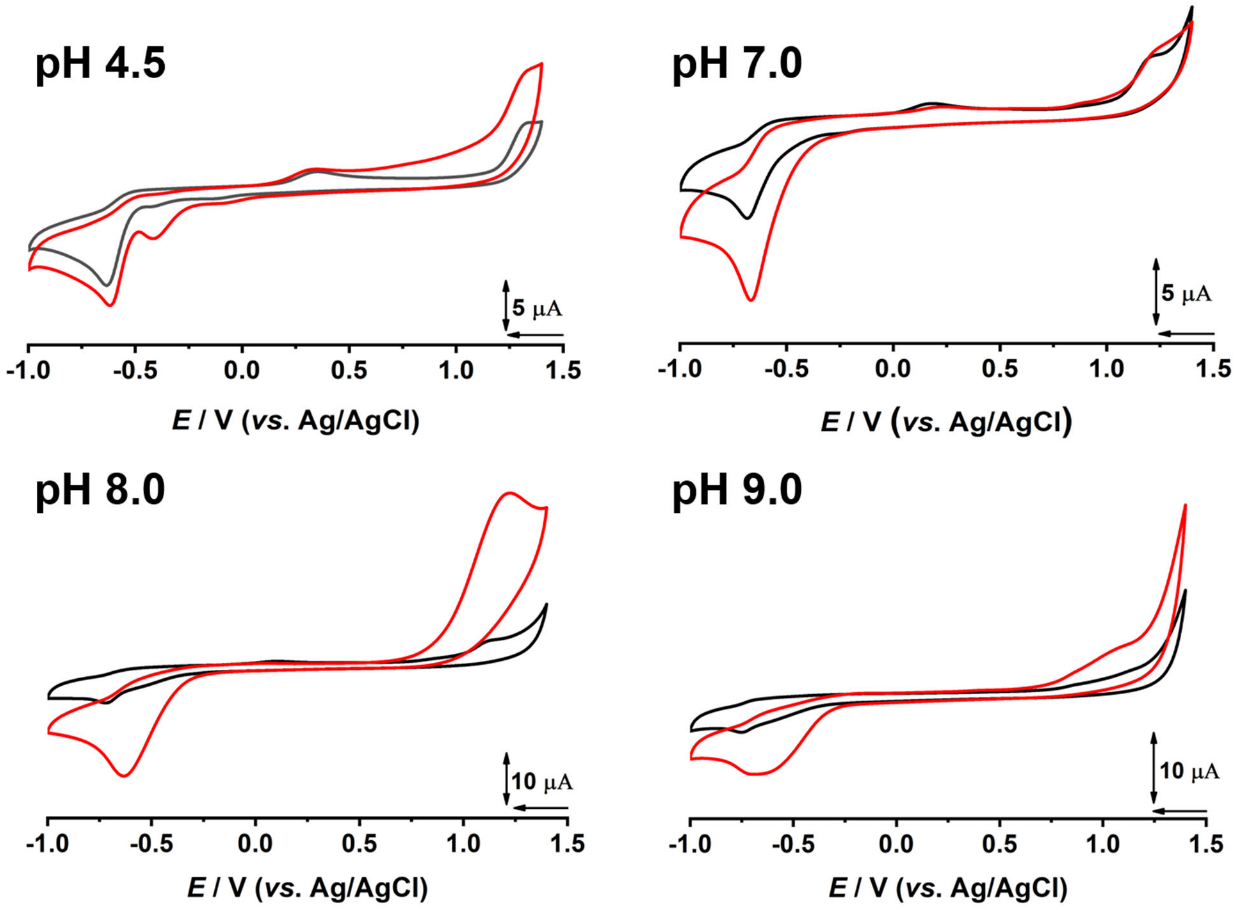

Figure S1. Cyclic voltammograms recorded for (▬) normal O_2 content and (▬) O_2 bubbled solutions of 500 μM AZA in 0.1 M buffer with different pHs; $\nu = 100\ mVs^{-1}$.

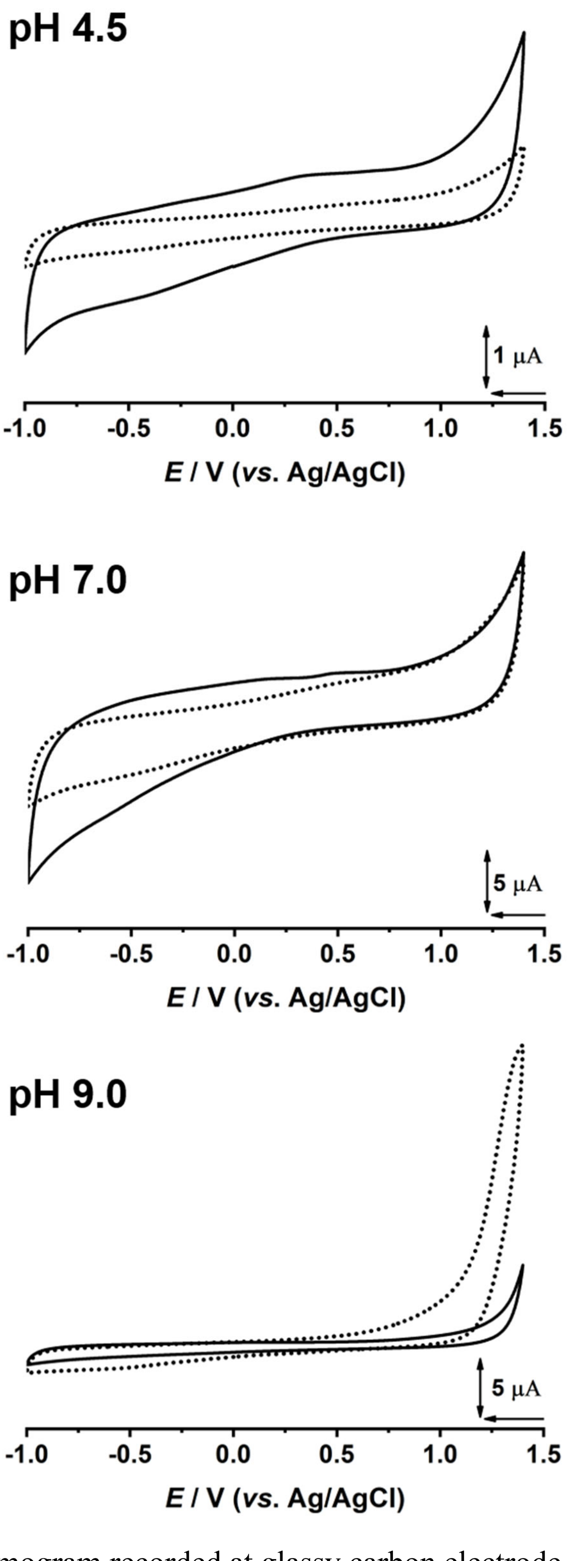

Figure S2. Cyclic voltammogram recorded at glassy carbon electrode in (▬) different pHs and (•••) 0.9 mM DMSO

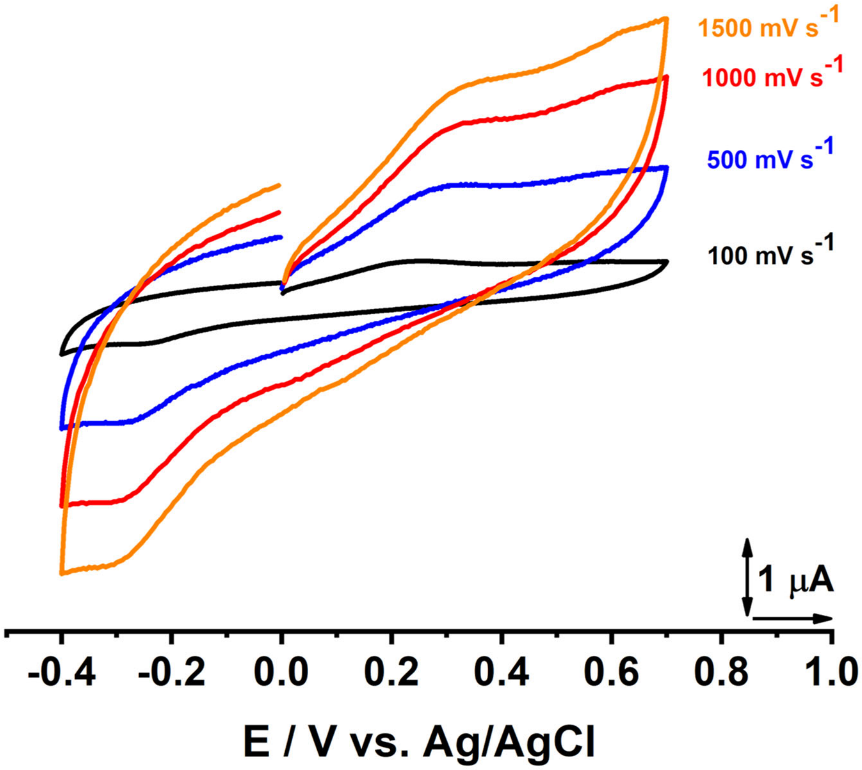

Figure.S3. Cyclic voltammogram recorded in 500 uM AZA after aplied potential 10 min from

E_0 =0 V, E_{min} =-0.4 V, E max=0.7 V(-0.6 V)

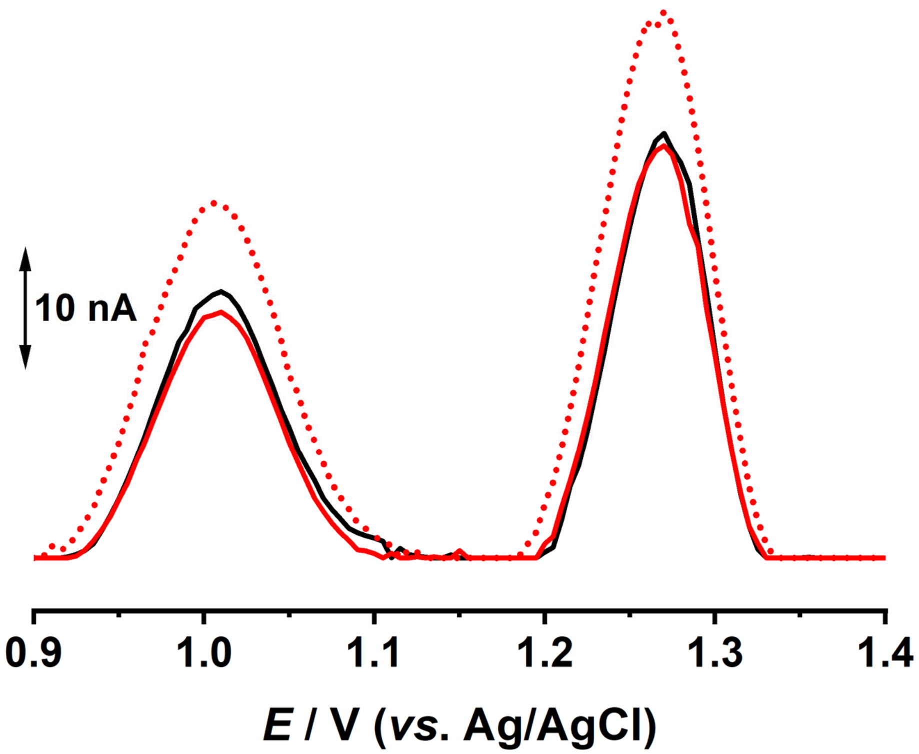

Figure.S4. DP voltammograms recorded for (▬) DNA biosensor without applied potential, (▬) DNA biosensor incubated in AZA (10 min) without applied potential and (●●●) DNA biosensor incubated in AZA (10 min) with applied potential.

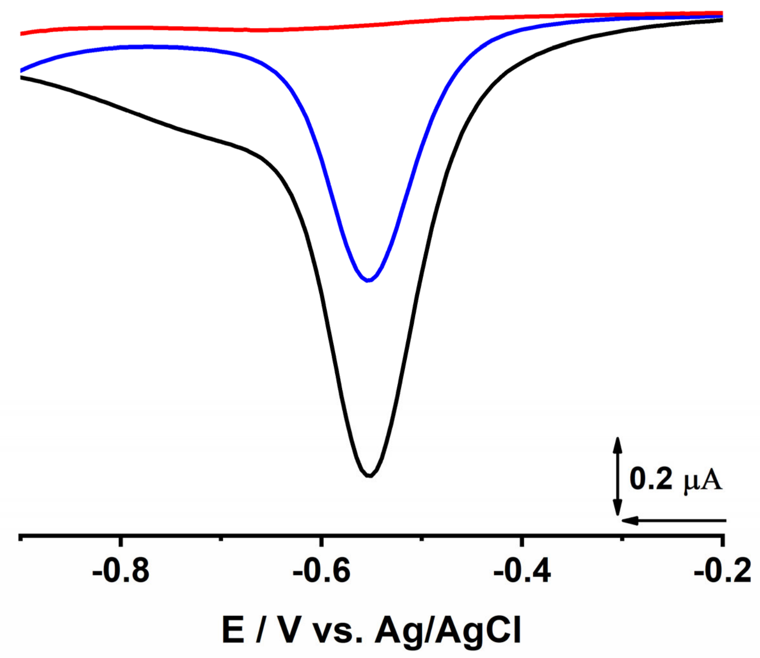

Figure S5. DP voltammograms recorded in acetate buffer pH 4.5 for DNA biosensor incubated with AZA for (▬) 10 min without applied potential, (▬) 10 min with applied potential and (▬) 20 min with applied potential.
